# LINC00907 suppresses malignant progression in hepatocellular carcinoma by inhibiting miR-24-3p

**DOI:** 10.1186/s41065-026-00714-6

**Published:** 2026-07-23

**Authors:** Jihua Cao, Weiyong Yang, Xiao Yang, Huayun Zhu

**Affiliations:** 1 Department of Hepatobiliary Surgery, The First College of Clinical Medical Science, China Three Gorges University, Yichang Central Peoples Hospital, Yichang, 443000 China; 2 Oncology Department, Xinchang Hospital of Chinese Medicine, Shaoxing, 312500 China; 3 Digestive Oncology Department, Changzhou Third Peoples Hospital, Changzhou, 213000 China; 4 Departments of Medical Oncology, Jiangsu Cancer Hospital & Institute Affiliated to Nanjing Medical University, No.42, Baiziting Road, Nanjing, 210009 China

**Keywords:** HCC, LINC00907, MiR-24-3p, Malignant phenotype, Clinical data

## Abstract

**Objective:**

This study aimed to clarify the role of LINC00907 in hepatocellular carcinoma (HCC) and its underlying molecular mechanisms.

**Methods:**

The research population comprised 117 participants diagnosed with HCC. The expressions of LINC00907 and miR-24-3p in both tissue specimens and cell lines, along with those of EMT markers and the MMP-2 and MMP-9, were measured using RT-qPCR. Cellular proliferation was measured with the CCK-8 assay, while apoptotic rates were determined by flow cytometry. The direct interactions between LINC00907, miR-24-3p, and PDGFRL were validated by dual luciferase reporter and RNA pull-down assays. HCC cells were transfected with constructs specifically modulating LINC00907 and miR-24-3p to investigate the impact of their expression on malignant phenotypic behaviors.

**Results:**

LINC00907 expression was significantly downregulated in HCC, whereas miR-24-3p expression was upregulated. Low LINC00907 expression was linked to significantly worse overall survival, as determined by Kaplan-Meier analysis. The upregulation of LINC00907 led to the suppression of cell proliferation and EMT, while inducing apoptosis and reducing the levels of MMP-2 and MMP-9. LINC00907 was shown to directly target and negatively regulate miR-24-3p. Furthermore, rescue experiments demonstrated that upregulation of miR-24-3p counteracted the tumor-suppressive effects mediated by LINC00907 overexpression in HCC cells.

**Conclusion:**

LINC00907 exerts tumor-suppressive effects by targeting and negatively regulating miR-24-3p, thereby influencing proliferation, apoptosis, and EMT in HCC cells.

**Graphical Abstract:**

**Figure:**
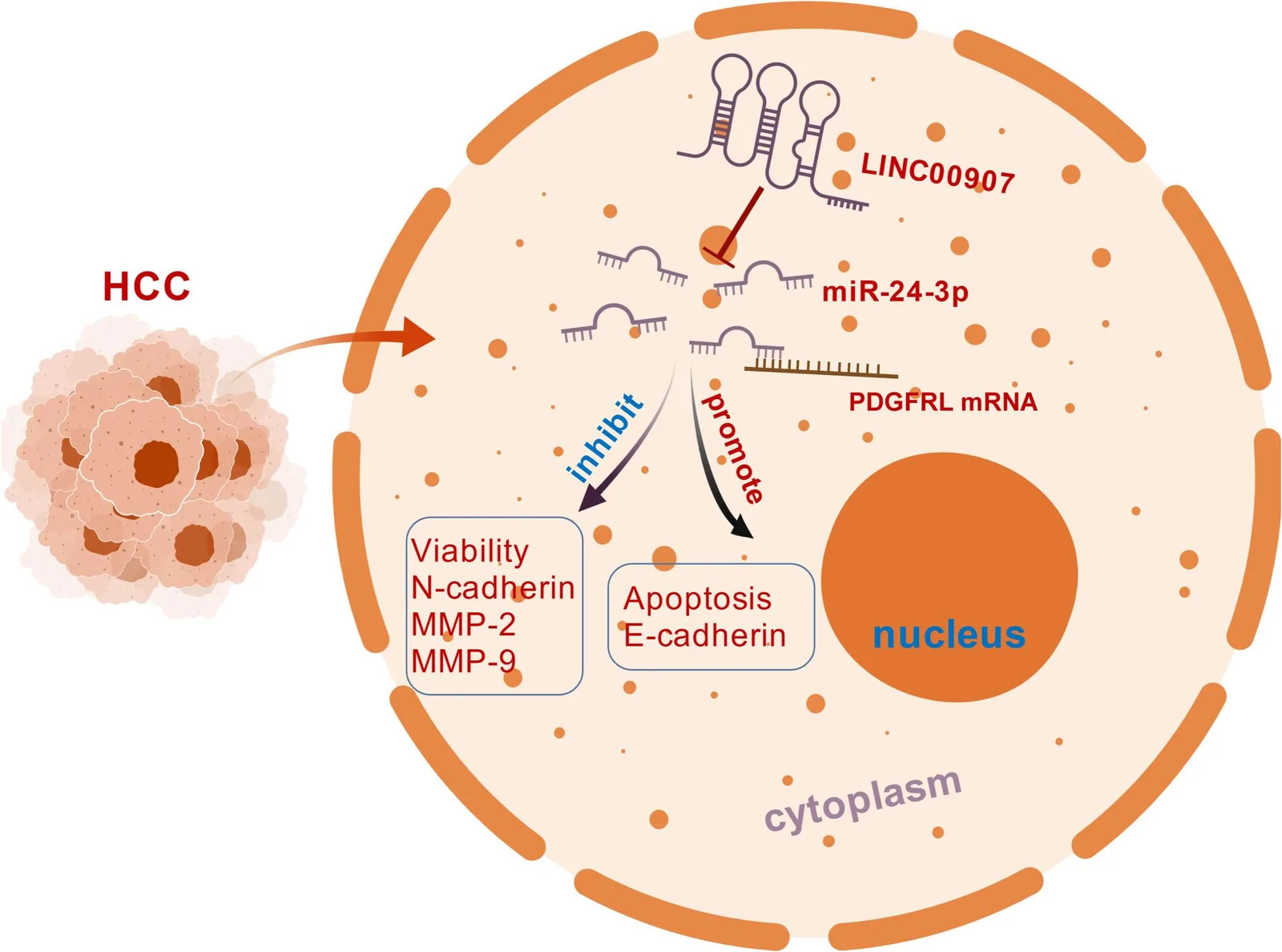

**Supplementary information:**

The online version contains supplementary material available at https://doi.org/10.1186/s41065-026-00714-6.

## Introduction

Hepatocellular carcinoma (HCC) is a prevalent primary liver cancer and a major cause of cancer deaths worldwide [1, 2]. This disease is particularly prevalent among individuals with chronic liver disease [3]. Current therapeutic options for HCC remain relatively limited, with poor prognosis for advanced-stage patients and overall survival rates that are far from encouraging [4–7]. Consequently, exploring the mechanisms behind HCC initiation and progression, along with the discovery of new prognostic biomarkers and therapeutic targets, holds significant clinical importance.

Due to their involvement in various diseases, long non-coding RNAs (lncRNAs) have become a hot research topic [8]. Studies have shown that LINC00907 is differentially expressed in HCC [9]. Metabolic dysfunction-associated steatohepatitis (MASH) is a potential precursor to HCC [10]. Furthermore, LINC00907 expression has been strongly linked to disease progression in NASH [11]. It also exhibits aberrant expression in the aortas of patients with thoracic aortic dissection (TAD) [12]. Furthermore, our analysis of public databases (TCGA, GEO) revealed that LINC00907 is significantly downregulated in HCC tissues, yet its regulatory mechanisms in HCC cells remain poorly understood. Therefore, this study selected LINC00907 as the research target to elucidate its role in the development and progression of HCC.

MicroRNAs (miRNAs) play a significant role in tumorigenesis by regulating gene expression. Among these, miR-24-3p has been frequently implicated in HCC. Depleting miR-24-3p levels curbs the process of epithelial-mesenchymal transition (EMT) in HCC cells [13]. Conversely, elevated levels of miR-24-3p promote the malignant progression of HCC cells [14]. Furthermore, overexpression of the MT1JP curbs HCC cell proliferation and migration by regulating miR-24-3p [15]. Notably, bioinformatic analyses suggest the existence of potential binding sites between LINC00907 and miR-24-3p. However, the regulatory relationship between these two molecules and their specific mechanisms in driving the malignant phenotypes of HCC remain unclear.

Based on this evidence, this study hypothesizes that LINC00907 may participate in HCC progression by sequestering miR-24-3p. The research aims to elucidate the mechanisms of LINC00907 in the malignant progression of HCC, thereby providing novel theoretical foundations and potential targets for molecularly targeted therapies for HCC.

## Materials and methods

### Patients and database

The study population consisted of 117 individuals who received a diagnosis of HCC and were managed at Changzhou Third People’s Hospital between July 2017 and July 2020. The HCC tumor samples and their corresponding adjacent non-tumorous tissues used in this study were obtained from surgically resected specimens. The inclusion criteria were as follows: a) confirmation of HCC through imaging and pathological biopsy; and without any prior chemotherapy or radiotherapy before their surgery. The exclusion criteria included: (a) the presence of other malignant tumors; (b) a history of liver transplantation; or (c) concomitant autoimmune diseases. All specimens were collected according to standardized protocols and stored at −80 °C. Furthermore, we retrieved the expression data of LINC00907 in HCC tumors from the GEPIA and UALCAN databases.

Ethical oversight for this study was provided by the Institutional Review Board (IRB) of Changzhou Third People’s Hospital, ensuring full compliance with the Declaration of Helsinki. Written informed consent was obtained from all individuals involved in the research.

### Culturing and transfection of cells

The normal hepatocyte line THLE-3 and the HCC lines Huh7 and Hep3B were sourced from ATCC. The Hep3B cells (with p53 deficiency and HBV genome integration) and the Huh7 cells (with wild-type p53 and negative HBV) form complementary molecular and genetic backgrounds, and thus were selected for the study of the mechanisms of HCC. Cell culture was performed using Dulbecco’s Modified Eagle Medium (DMEM) (Gibco, USA) enriched with 10% fetal bovine serum (FBS) at 37°C and 5% CO2. To generate an LINC00907 overexpression model, its full-length sequence was inserted into the pcDNA3.1 vector (Invitrogen, USA), resulting in the recombinant plasmid denoted as pcDNA3.1-LINC00907. The small interfering RNA of LINC00907 (si-LINC00907) and the corresponding negative control (si-NC) were provided by RiboBio Biotechnology Company (China). Lipofectamine™ 2000 reagent (Invitrogen, USA) was used for transfections. The miR-24-3p mimics, NC mimics, the empty pcDNA3.1 vector, and the LINC00907 overexpression plasmid (pcDNA3.1-LINC00907) were individually transfected into Huh7 and Hep3B cells. All subsequent functional assays were performed 48 hours after transfection.

### RT-qPCR

Total RNA from clinical tissue samples and cells was extracted utilizing TRIzol™ reagent (Invitrogen, USA), adhering to the supplier’s guidelines. The synthesis of complementary DNA (cDNA) was carried out by employing the PrimeScript RT Kit (Takara, Japan). RT-qPCR was carried out using SYBR Green Premix Ex Taq™ (Takara, Japan) on an ABI PRISM 7000 Flash Thermal Analyser (Applied Biosystems, USA). GAPDH served as an endogenous control for LINC00907 and the mRNAs of E-cadherin, N-cadherin, MMP-2, and MMP-9, whereas U6 functioned to normalize miRNA expressions. To evaluate relative gene expression, the 2^−ΔΔCt^ method was applied.

### Cell proliferation assay

Following the respective treatments, the 96-well plates were seeded with cells at 2 × 10^3^ cells per well. At each prescribed time point (0, 24, 48, and 72 h), treat the cells in with 10 µL of CCK-8 reagent (Dojindo, Japan). A Thermo Fisher Scientific (USA) microplate reader was used to measure the absorbance at 450 nm following incubation at 37 °C for one hour.

### Apoptosis detection

After transfection, cells were collected by trypsinization. After being washed twice with phosphate-buffered saline (PBS), the cell pellet was resuspended in 100 µL of 1× binding buffer at a density of 1 × 10^6^ cells/mL. Subsequently, 5 µL of Annexin V-FITC (Thermo Fisher Scientific, USA) and 5 µL of propidium iodide (PI) (Thermo Fisher Scientific, USA) were added to the cell suspension, with incubation then proceeding for 5 min under dark conditions. Assessment of apoptosis was carried out by flow cytometry using a FACScan instrument (Becton Dickinson, USA).

### Subcellular localization analysis

To investigate the subcellular distribution of LINC00907, nuclear and cytoplasmic fractions were isolated from HCC cells using a Cytoplasmic & Nuclear RNA Purification Kit (Norgen Biotek, Canada) as stipulated in the manufacturer’s protocol. RNA from each fraction was subjected to RT-qPCR to quantify LINC00907 expression.

### Dual luciferase reporter (DLR) assay

Potential target gene relationships were predicted by the LncRNASNP2 database. The PrimGLO-LINC00907/PDGFRL-wt and PrimGLO-LINC00907/PDGFRL-mut reporter plasmids were constructed and subsequently co-introduced into cells alongside either a miR-24-3p mimic or a negative control. Forty-eight hours post-transfection, luminescence signals were assessed with the DLR Assay System (Promega, USA), following the supplier’s recommended procedures.

### RNA pull-down

Magnetic beads functionalized with biotin-labeled miR-24-3p probes were incubated with lysates derived from Hep3B and Huh7 cells that had been transfected with miR-24-3p mimics. Subsequently, the biotin-labeled RNA complex was precipitated using the RNeasy Mini Kit (QIAGEN, Germany). The precipitated RNA was subsequently analyzed via RT-qPCR.

### Statistical analysis

Data was analyzed using SPSS 20.0 software and visualized with GraphPad Prism version 9.0. Student’s t-test was employed for pairwise comparisons, whereas one-way/two-way analysis of variance (ANOVA) was used for multiple comparisons. A statistical significance threshold of *P* < 0.05 was adopted. All experimental findings were generated from a minimum of three independent replicates, with values reported as the mean ± standard deviation.

### Culturing and transfection of cells

The normal hepatocyte line THLE-3 and the HCC lines Huh7 and Hep3B were sourced from ATCC. The Hep3B cells (with p53 deficiency and HBV genome integration) and the Huh7 cells (with wild-type p53 and negative HBV) form complementary molecular and genetic backgrounds, and thus were selected for the study of the mechanisms of HCC. Cell culture was performed using Dulbecco’s Modified Eagle Medium (DMEM) (Gibco, USA) enriched with 10% fetal bovine serum (FBS) at 37 °C and 5% CO_2_. To generate an LINC00907 overexpression model, its full-length sequence was inserted into the pcDNA3.1 vector (Invitrogen, USA), resulting in the recombinant plasmid denoted as pcDNA3.1-LINC00907. The small interfering RNA of LINC00907 (si-LINC00907) and the corresponding negative control (si-NC) were provided by RiboBio Biotechnology Company (China). Lipofectamine™ 2000 reagent (Invitrogen, USA) was used for transfections. The miR-24-3p mimics, NC mimics, the empty pcDNA3.1 vector, and the LINC00907 overexpression plasmid (pcDNA3.1-LINC00907) were individually transfected into Huh7 and Hep3B cells. All subsequent functional assays were performed 48 h after transfection.

## Results

### LINC00907 expression levels in HCC tissues and their association with patient prognosis

**Fig. 1:**
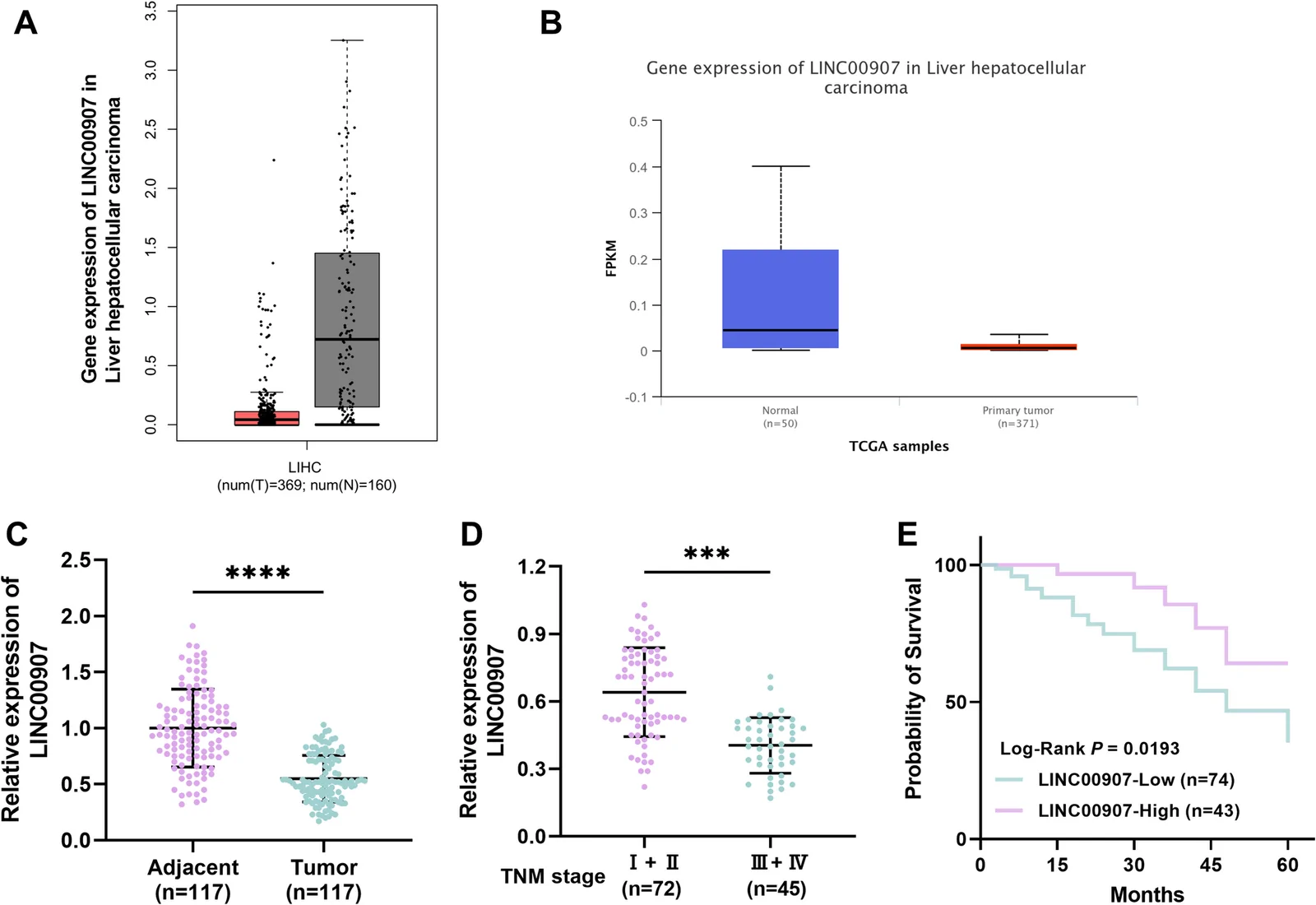
LINC00907 was found to exhibit low expression in HCC tissues and to be associated with poor prognosis. **A**-**B** LINC00907 expression was analyzed in the TCGA dataset using the GEPIA and UALCAN online tools. **C** The expression levels of LINC00907 were detected in 57 pairs of tumors and adjacent non-cancerous tissues by RT-qPCR. **D** Expression of LINC00907 in HCC tumors with different TNM stages. **E** A Kaplan-Meier analysis was performed to assess the correlation between LINC00907 expression levels and patient overall survival. ***P < 0.001, ****P < 0.0001

Analysis of the GEPIA and UALCAN databases revealed aberrant expression of LINC00907 in HCC samples (Fig. 1A-B). A comparative analysis of tumor and adjacent non-tumor tissues from 117 HCC patients demonstrated significantly lower LINC00907 expression in the cancerous tissues (*P* < 0.0001, Fig. 1C). Moreover, as the tumor node metastasis (TNM) stage increases, the histological level of LINC00907 decreases (*P* < 0.001, Fig. 1D). Stratification of HCC patients into high- and low-LINC00907 expression cohorts, based on the mean expression, was performed to investigate the relationship with their clinicopathological parameters. Diminished expression of LINC00907 was strongly tied to raised AFP concentrations, progression to later TNM stages, and the occurrence of lymph node metastasis. Conversely, it showed no meaningful connection to characteristics like age, gender, tumor size, or hepatitis background (*P* >0.05, Table 1). Accordingly, a significant difference in overall survival was observed via Kaplan-Meier analysis, with the low LINC00907 expression group faring worse than the high-expression group (*P* = 0.0193, Fig. 1E). Reduced LINC00907 expression served as an independent risk factor for diminished overall survival (*P* = 0.023, HR = 0.288, Table 2).

**Table 1:** The relationship between LINC00907 expression and clinical parameters in HCC

Characteristics	LINC00907 expression		*P*
	Low (*n* = 74)	High (*n* = 43)	
Age, years			0.435
< 55	33	16	
≥ 55	41	27	
Gender			0.340
Male	36	17	
Female	38	26	
Tumor size, cm			0.169
< 5	35	26	
≥ 5	39	17	
Combined with hepatitis			0.217
Yes	38	17	
No	36	26	
AFP, ng/mL			**0.032**
< 200	33	28	
≥ 200	41	15	
TNM stage			**0.029**
I + II	40	32	
III + IV	34	11	
Lymph node metastasis			**0.042**
Yes	29	9	
No	45	34	

*AFP* Alpha-fetoprotein; *TNM* tumor node metastasis

**Table 2:** Multivariate Cox analysis of LINC00907 and clinical parameters with overall survival

Characteristics	Multivariate analysis		
	*P*	HR	95%CI
LINC00907	0.023	0.288	0.099–0.840
Age	0.068	2.064	0.947–4.499
Gender	0.071	0.494	0.230–1.061
Tumor size	0.121	1.929	0.841–4.426
Combined with hepatitis	0.572	1.241	0.587–2.622
AFP	0.039	2.542	1.050–6.150
TNM stage	0.034	2.533	1.072–5.986
Lymph node metastasis	0.047	2.458	1.011–5.977

*AFP* Alpha-fetoprotein; *TNM* tumor node metastasis

### Expression and localization analysis of LINC00907 in HCC cells

**Fig. 2:**
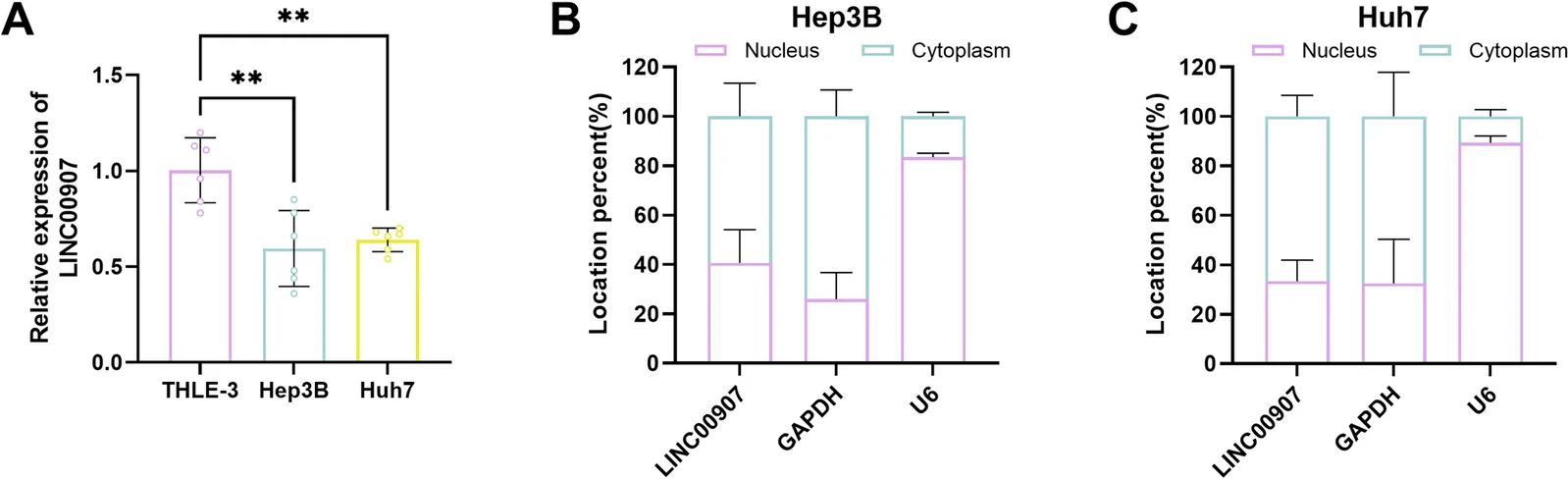
LINC00907 was downregulated in HCC cell lines and was primarily localized in the cytoplasm. **A** The expression level of LINC00907 was detected in the human normal hepatocyte cell line (THLE-3) and HCC cell lines (Hep3B and Huh7) using RT-qPCR. Subcellular localization of LINC00907 was determined in Hep3B (**B**) and Huh7 (**C**) cells using nuclear-cytoplasmic fractionation assays. ** *P* < 0.01

Further assessment of LINC00907 expression via RT-qPCR in HCC cells revealed significantly lower levels in the HCC cells (Hep3B, Huh7) relative to the normal hepatocyte cell line (THLE-3) (*P* < 0.01, Fig. 2A). Analysis of the subcellular localization of LINC00907 revealed its predominant localization to the cytoplasm of these cancer cell lines (Figs. 2B-C). This localization pattern suggests that LINC00907 could exert its regulatory functions through sequestering cytoplasmic miRNAs.

### Upregulation of LINC00907 influences the biological behavior of HCC cells

**Fig. 3:**
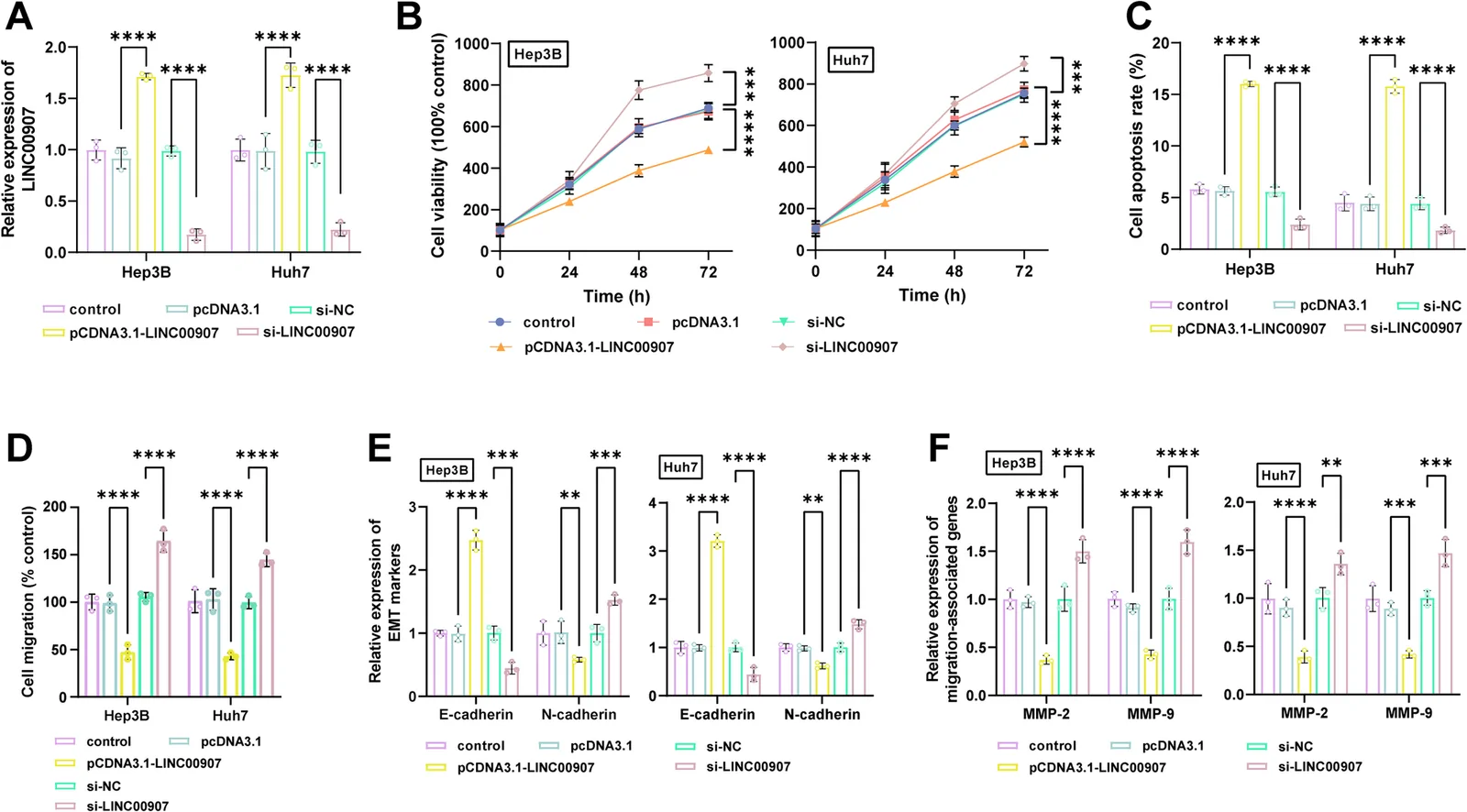
Role of LINC00907 in HCC cell proliferation, apoptosis, and migration. **A** RT-qPCR analysis of pCDNA3.1-LINC00907/si-LINC00907 transfection efficiency. **B** CCK-8 assay evaluating the effects of LINC00907 on cell proliferation. **C** Flow cytometry analysis of the effect of LINC00907 overexpression or silencing on HCC cell apoptosis. **D** The Transwell assay served to investigate the effect of LINC00907 on the migration ability of HCC cells. **E** RT-qPCR analysis of the effects of LINC00907 overexpression/silencing on the mRNA expression levels of EMT markers (E-cadherin and N-cadherin). **F** RT-qPCR analysis of the effects of LINC00907 overexpression/silencing on the mRNA expression levels of migration-related genes (MMP-2 and MMP-9). ** *P* < 0.01, ***P < 0.001, ****P < 0.0001

To elucidate the role of LINC00907 in HCC, we quantified its levels via RT-qPCR to assess the efficiency of transfection in Huh7 and Hep3B. Compared with the control group, the expression of LINC00907 in the pcDNA3.1-LINC00907 transfection group was significantly increased, while in the si-LINC00907 transfection group, its expression was effectively silenced (*P* < 0.0001, Fig. 3A). Subsequent functional assays demonstrated that upregulating LINC00907 markedly suppressed the proliferation of HCC cells and promoted apoptosis, while silencing LINC00907 had the opposite effect (*P* < 0.0001, Fig. 3B-C). The invasive ability of HCC cells was inhibited by overexpression of LINC00907, while it was enhanced by knockdown of LINC00907 (*P* < 0.0001, Fig. 3D). Concurrently, in LINC00907-overexpressed cells, E-cadherin expression was upregulated (*P* < 0.0001, Fig. 3E), while N-cadherin expression was downregulated (*P* < 0.01, Fig. 3E). After knocking down LINC00907, the expression of E-cadherin was inhibited while the expression of N-cadherin was promoted (*P* < 0.001, Fig. 3E). Moreover, MMP-2 and MMP-9 expressions were significantly reduced by LINC00907 and were suppressed by its silencing (*P* < 0.0001, Fig. 3F). These results demonstrate that LINC00907 may inhibit the malignant phenotype of HCC cells.

### MiR-24-3p is directly targeted and sponged by LINC00907

**Fig. 4:**
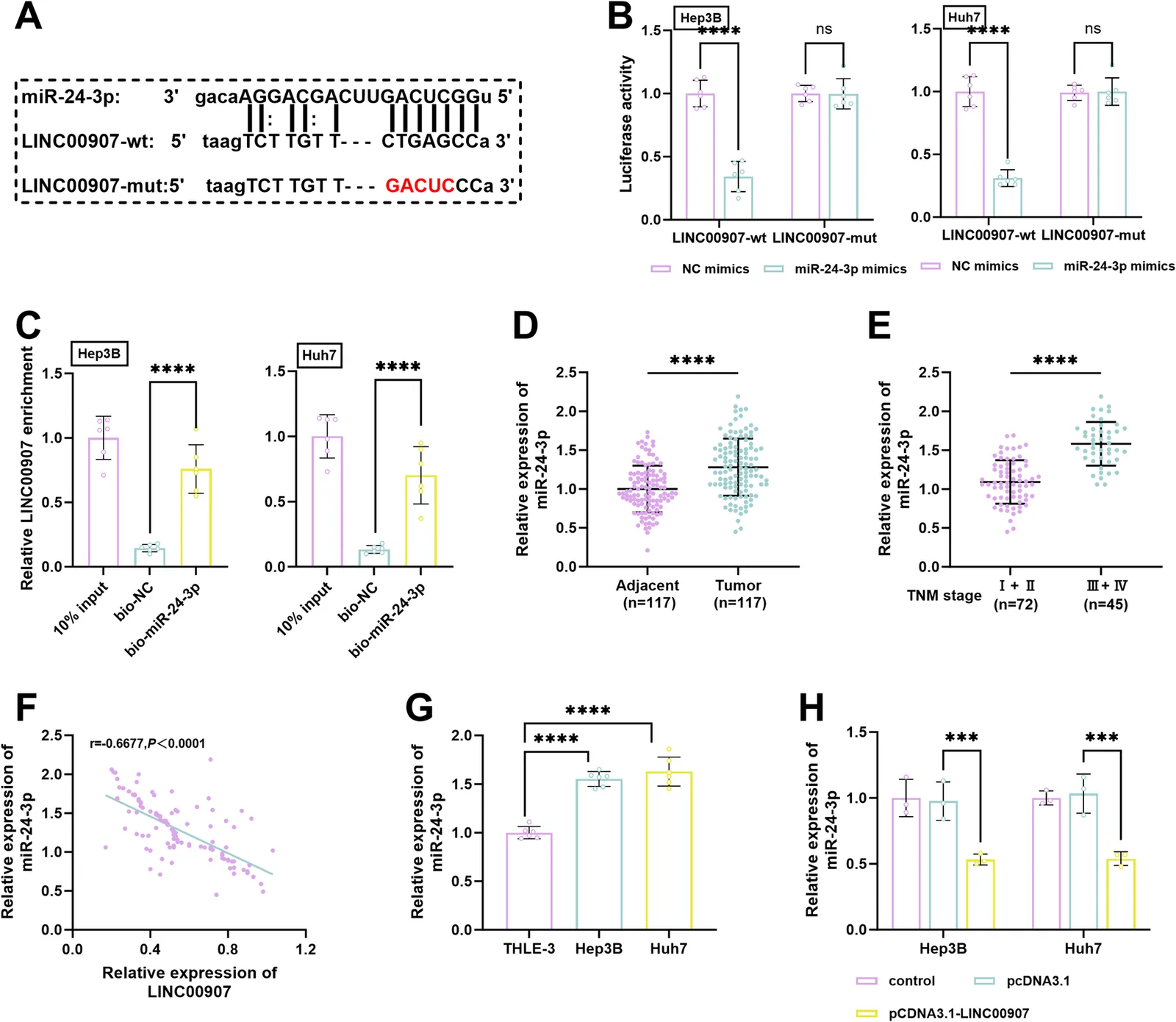
miR-24-3p is a downstream target of LINC00907. **A** Prediction of potential binding sites between LINC00907 and miR-24-3p using the LncRNASNP2 database. **B** Measurement of luciferase activity in Hep3B and Huh7 cells transfected with either NC mimics or miR-24-3p mimics. **C** RNA pull-down assay validation of the binding between LINC00907 and miR-24-3p. **D** RT-qPCR quantification of miR-24-3p expression levels in HCC tissues. **E** The expression of miR-24-3p in HCC tumors of different TNM stages. **F** Pearson correlation analysis of LINC00907 and miR-24-3p expression levels in HCC tissues. **G** RT-qPCR quantification of miR-24-3p expression levels in HCC cell lines. **H** Assessment of the effect of LINC00907 overexpression on miR-24-3p expression levels by RT-qPCR. *** *P* < 0.001, **** *P* < 0.0001

An analysis using the LncRNASNP2 online database identified a potential binding sequence between LINC00907 and miR-24-3p (Fig. 4A). DLR assays further confirmed that co-transfection of miR-24-3p mimics with the LINC00907-wt resulted in a marked decrease in luciferase activity (*P* < 0.0001, Fig. 4B), but co-transfection with the LINC00907-mut did not cause a significant change (*P* >0.05, Fig. 4B). Moreover, RNA pull-down assays demonstrated that LINC00907 specifically binds to biotin-labeled miR-24-3p probes (*P* < 0.0001, Fig. 4C). miR-24-3p levels were markedly elevated in comparison to non-tumorous tissues in HCC tissues (*P* < 0.0001, Fig. 4D). In tumors with high TNM grades, the expression of miR-24-3p further increased (*P* < 0.0001, Fig. 4E). According to the evaluation by Pearson correlation, the levels of LINC00907 and miR-24-3p in HCC revealed an inverse association that was statistically significant (*r* = − 0.6677, *P* < 0.0001, Fig. 4F). Similarly, a marked elevation in miR-24-3p expression was observed in HCC cells (*P* < 0.0001, Fig. 4G). Furthermore, overexpression of LINC00907 in HCC cells significantly suppressed miR-24-3p levels (*P* < 0.001, Fig. 4H).

### LINC00907 inhibits malignant progression in HCC by targeting miR-24-3p

**Fig. 5:**
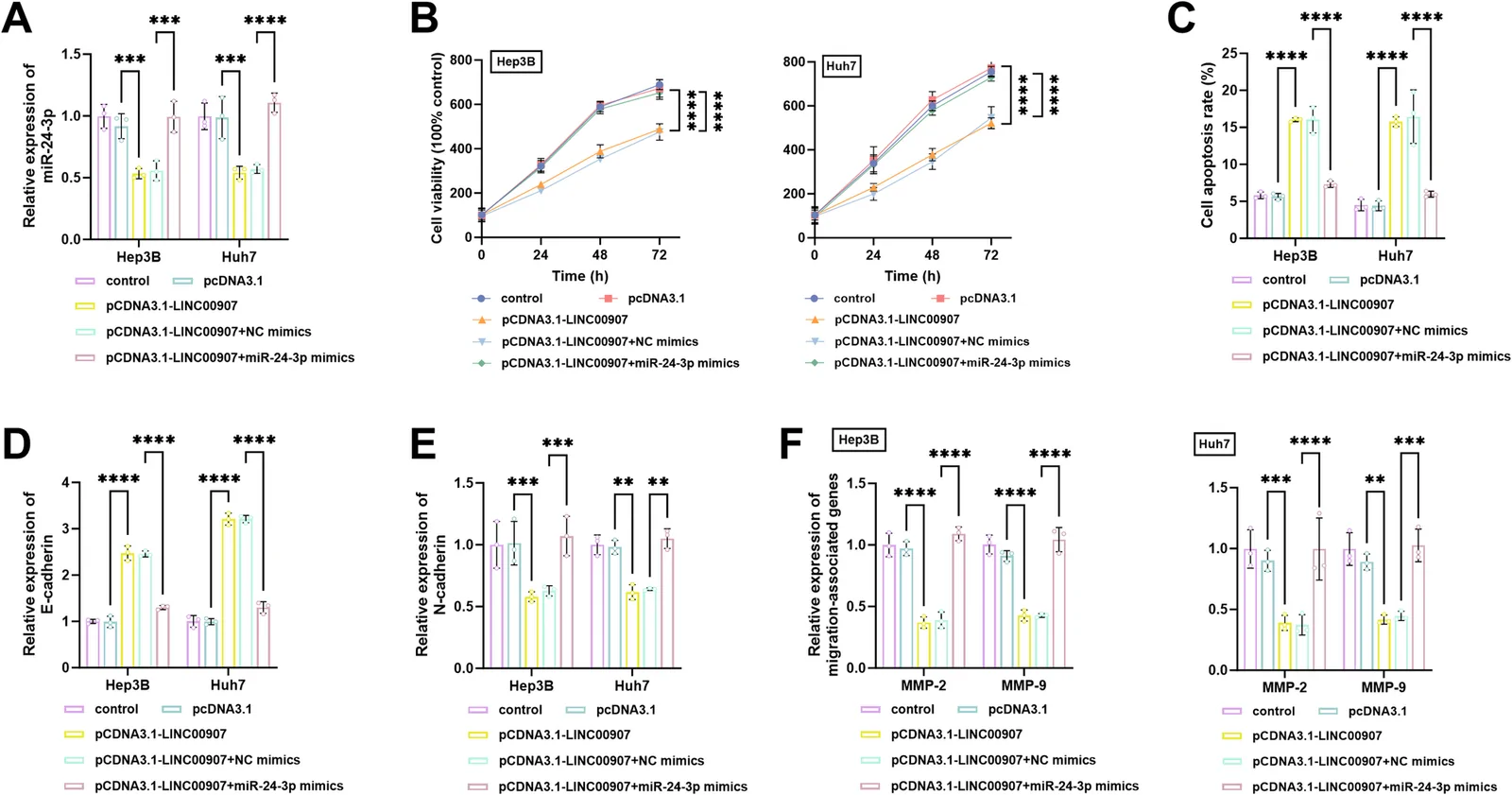
LINC00907 inhibits proliferation, migration, and EMT in HCC cells by targeting miR-24-3p. **A** miR-24-3p expression levels were detected by RT-qPCR after different transfection treatments. **B** Cell proliferation was assessed by CCK-8 assay after different transfection treatments. **C** Apoptosis of HCC cells were evaluated by flow cytometry after different transfection treatments. The mRNA expression levels of the EMT markers E-cadherin (**D**) and N-cadherin (**E**) were measured by RT-qPCR. **F** The mRNA expression levels of the migration-related proteins MMP-2 and MMP-9 were detected by RT-qPCR. ** *P* < 0.01, *** *P* < 0.001, **** *P* < 0.0001

To investigate how LINC00907 participates in HCC progression, we introduced LINC00907 overexpression vector into HCC cells, followed by transfection with miR-24-3p mimics. The results demonstrated that the introduction of the mimics markedly reversed the reduction in miR-24-3p expression caused by LINC00907 overexpression (*P* < 0.001, Fig. 5A). Upregulation of miR-24-3p rescued the LINC00907-induced restriction of HCC cell proliferation and promotion of apoptosis (*P* < 0.0001, Fig. 5B-C). Similarly, adding miR-24-3p counteracted the effect of LINC00907 overexpression on EMT markers. Specifically, it attenuated the LINC00907-induced increase in E-cadherin and decrease in N-cadherin (*P* < 0.01, Fig. 5D-E). Furthermore, overexpression of miR-24-3p attenuated the inhibition of MMP-2 and MMP-9 expression by LINC00907 (*P* < 0.01, Fig. 5F). These findings indicate that LINC00907 may inhibit HCC progression by targeting miR-24-3p.

### PDGFRL is a direct downstream target of miR-24-3p

**Fig. 6:**
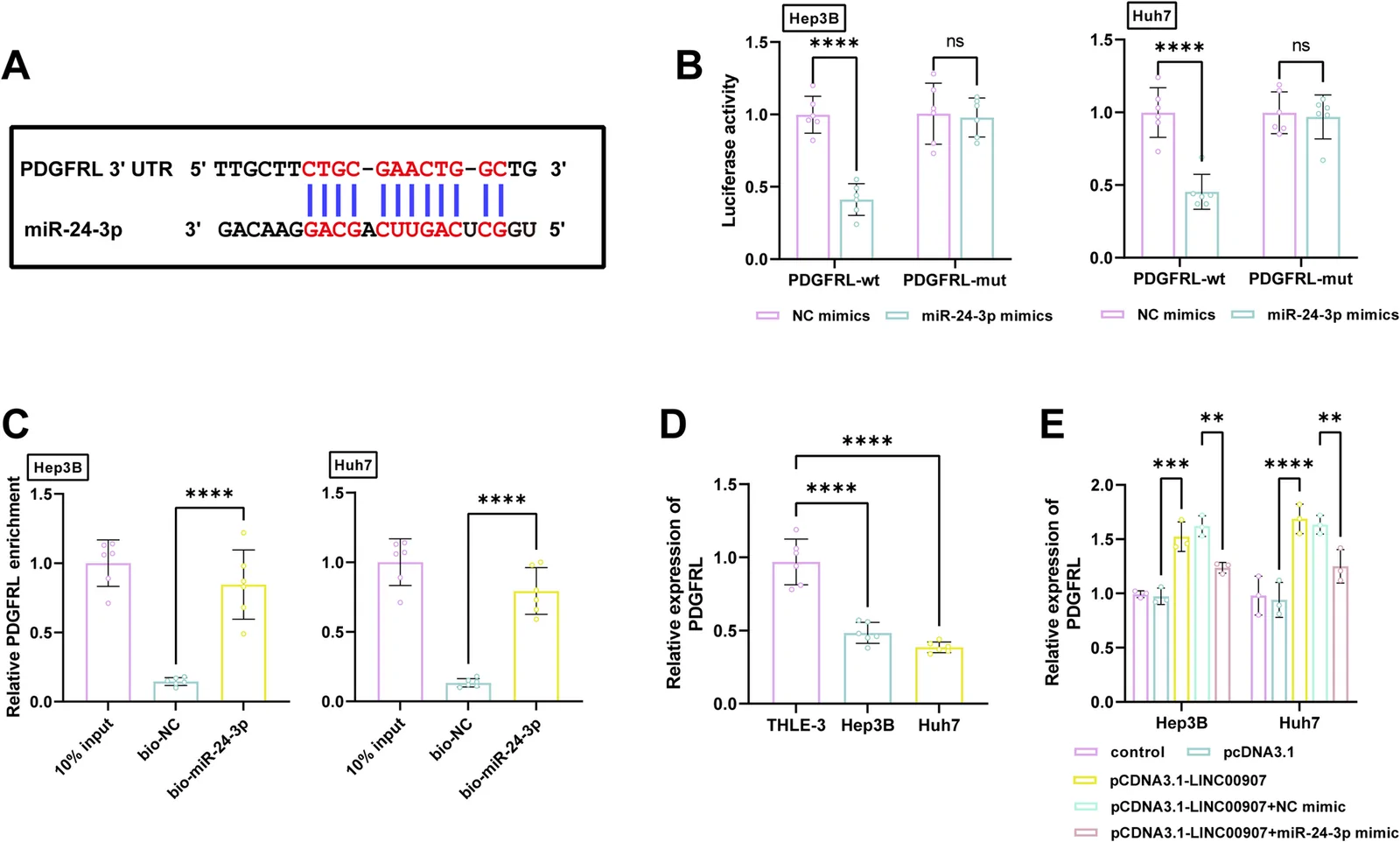
miR-24-3p targets PDGFRL. **A** Potential binding sites of miR-24-3p in the 3’UTR of PDGFRL. **B-C** DLR and RIP experiments in hepatocellular carcinoma cells validate their interaction. **D** Expression of PDGFRL in HCC cell lines. **E** Effects of different transfection treatments on PDGFRL expression in HCC cell lines. ** *P* < 0.01, *** *P* < 0.001, **** *P* < 0.0001

We utilized the miRWalk database to predict PDGFRL as a downstream target of miR-24-3p (Fig. 6A). To validate this targeting relationship, a DLR assay was performed in HCC cells. Results showed that the miR-24-3p mimics significantly reduced the luciferase activity of PDGFRL-wt (*P* < 0.0001, Fig. 6B), while having no significant effect on PDGFRL-mut (*P* > 0.05, Fig. 6B). RNA pull-down experiments confirmed that PDGFRL specifically binds to biotin-labeled miR-24-3p probes (*P* < 0.0001, Fig. 6C), further supporting their interaction. Compared to normal cells, PDGFRL expression was markedly reduced in HCC cells (*P* < 0.0001, Fig. 6D). Furthermore, PDGFRL expression increased upon LINC00907 overexpression, while miR-24-3p mimic transfection significantly reversed the PDGFRL upregulation induced by LINC00907 overexpression (*P* < 0.01, Fig. 6E).

## Discussion

HCC is a highly prevalent malignant liver tumor worldwide [16]. Recent studies have demonstrated that lncRNAs exert crucial regulatory functions in the pathogenesis of multiple solid tumors, including HCC [17–19]. LINC00907, located on chromosome 18, exhibits significantly altered expression in HCC. However, its specific molecular mechanisms underlying HCC initiation and progression remain unclear. Our experimental data demonstrate that LINC00907 expression is markedly decreased in HCC. A decrease in its expression was closely correlated with multiple adverse clinical features in patients, including elevated AFP, advanced TNM classification, and lymphatic spread. It was also confirmed to be a risk factor for reduced overall survival. This conclusion is consistent with previous studies demonstrating that certain tumor-suppressing lncRNAs (e.g., MEG3 [20], LIMT[21]) are downregulated in HCC and are associated with poor prognosis, further corroborating the crucial regulatory role of lncRNAs in HCC progression.

Cell death represents an orderly and autonomous process of terminating cellular life activities under specific physiological or pathological conditions. Dysregulation of this process can disrupt the normal cell growth cycle, thereby contributing to various disease states [15, 22, 23]. Non-coding RNAs modulate cellular proliferation and differentiation by regulating cell death, thereby playing a pivotal part in the onset and progression of numerous diseases [24, 25]. Migration, as a fundamental cellular function, is critically involved in tumor pathological conditions [26]. Metastasis, a process wherein the EMT confers enhanced migratory and invasive potential on tumor cells. This core process thereby facilitates the invasion of tumor cells into the stroma and their dissemination to distant sites [27, 28]. In this study, overexpression of LINC00907 significantly suppressed the proliferation of HCC cells and the EMT process, while simultaneously promoting apoptosis and downregulating MMP-2 and MMP-9 levels. Collectively, LINC00907 has potential anti-cancer effects in HCC.

Previous research has established that miR-24-3p acts as an oncogenic microRNA, promoting tumor progression in colorectal cancer [29] and lung cancer [30]. Our investigation noted a marked upregulation of miR-24-3p expression in HCC tissues and corresponding cell lines, which suggested that miR-24-3p may represent a potential therapeutic target in liver cancer. Notably, lncRNAs can function as ‘molecular sponges’ that sequester endogenous miRNAs, thereby participating in the regulation of tumorigenesis [31]. Subcellular localization analysis revealed that LINC00907 is predominantly localized in the cytoplasm, suggesting its potential involvement in post-transcriptional regulation. Predictions from the LncRNASNP2 database indicated potential direct binding between LINC00907 and miR-24-3p, an interaction that was subsequently validated using a DLR assay. RNA pull-down experiments further demonstrated significant enrichment of miR-24-3p, confirming that LINC00907 acts as a competitive endogenous RNA (ceRNA) by sponging miR-24-3p. Additionally, LINC00907 and miR-24-3p exhibit a negative regulatory relationship, suggesting a functional association between them. Finally, functional rescue assays demonstrated that adding miR-24-3p significantly reversed the effects mediated by LINC00907, including the inhibition of proliferation, promotion of apoptosis, reversal of EMT, and downregulation of MMP-2 and MMP-9. These findings confirm that LINC00907 primarily exerts its anticancer effects by sequestering miR-24-3p.

PDGFRL is structurally similar to the PDGF receptor. Although PDGFRL lacks intrinsic kinase activity and does not bind to PDGF ligands or be activated by the classical growth factors, its imbalance is associated with the progression of HCC, possibly through protein-protein interactions or other regulatory pathways. It is reported that PDGFRL not only inhibits the proliferation of HCC cells, but also induces cancer cell apoptosis by blocking the STAT3 pathway. Furthermore, the upregulation of PDGFRL is considered to be the key mechanism by which regorafenib exerts its anti-tumor effect in HCC [32]. The miRWalk prediction revealed potential binding sites for miR-24-3p within the PDGFRL 3’UTR region. Subsequent DLR and RIP assays confirmed PDGFRL as a direct downstream target of miR-24-3p. Expression rescue experiments further demonstrated that LINC00907 upregulates PDGFRL expression via sponging miR-24-3p, suggesting LINC00907 may participate in HCC progression regulation through this molecular pathway.

In summary, our research elucidates the potential role of the lncRNA LINC00907 in HCC progression. Specifically, the low expression of LINC00907 is a marker of poor prognosis in HCC. Its overexpression may exert the function of suppressing cancer cell proliferation, migration, and inducing cell death through the miR-24-3p/PDGFRL axis (Supplementary Fig. 1). This discovery establishes a theoretical foundation for developing prognostic biomarkers and therapeutic targets.

## Supplementary information

Supplementary Material 1.

## Data Availability

The datasets used and/or analysed during the current study are available from the corresponding author on reasonable request.
